# A Spiking Neural Network Model of Model-Free Reinforcement Learning with High-Dimensional Sensory Input and Perceptual Ambiguity

**DOI:** 10.1371/journal.pone.0115620

**Published:** 2015-03-03

**Authors:** Takashi Nakano, Makoto Otsuka, Junichiro Yoshimoto, Kenji Doya

**Affiliations:** 1 Neurobiology Research Unit, Okinawa Institute of Science and Technology, 1919-1, Tancha, Onna-Son, Kunigami, Okinawa 904-0495 Japan; 2 Neural Computation Unit, Okinawa Institute of Science and Technology, 1919-1, Tancha, Onna-Son, Kunigami, Okinawa 904-0495 Japan; Plymouth University, UNITED KINGDOM

## Abstract

A theoretical framework of reinforcement learning plays an important role in understanding action selection in animals. Spiking neural networks provide a theoretically grounded means to test computational hypotheses on neurally plausible algorithms of reinforcement learning through numerical simulation. However, most of these models cannot handle observations which are noisy, or occurred in the past, even though these are inevitable and constraining features of learning in real environments. This class of problem is formally known as partially observable reinforcement learning (PORL) problems. It provides a generalization of reinforcement learning to partially observable domains. In addition, observations in the real world tend to be rich and high-dimensional. In this work, we use a spiking neural network model to approximate the free energy of a restricted Boltzmann machine and apply it to the solution of PORL problems with high-dimensional observations. Our spiking network model solves maze tasks with perceptually ambiguous high-dimensional observations without knowledge of the true environment. An extended model with working memory also solves history-dependent tasks. The way spiking neural networks handle PORL problems may provide a glimpse into the underlying laws of neural information processing which can only be discovered through such a top-down approach.

## Introduction

When faced with a novel environment, animals learn what actions to make through trial and error. Such reward driven learning with incomplete knowledge of the environment is called reinforcement learning (RL) [1]. Starting from prominent experimental findings which show that reward prediction errors are correlated with dopamine signals [2], many studies have investigated how reinforcement learning algorithms are implemented in the brain [3–5].

Numerical simulations of spiking neural networks (SNN) can be used to test whether reward learning algorithms are neurally plausible and to theoretically investigate the validity of computational hypotheses. There have been several successful implementations of reinforcement learning in SNNs [6–11].

However in many real world situations, the problems animals are faced with are more challenging than those that can be solved with RL. Observations are usually noisy and stochastic and optimal decision making often depends on past experience. The generalization of RL to such partially observable domains is known as partially observable reinforcement learning (PORL) [12]. The PORL problems can be divided into two subclasses depending on the task difficulty. When the optimal policy depends on the current observation, we call this case a history-independent PORL problem. On the other hand, when the optimal policy depends on the past observations, we call this case a history-dependent PORL problem. PORL problems provide a framework for solving partially observable Markov decision processes (POMDP) without full knowledge of the environment. It is firmly grounded theoretically and also general enough to model the decision making of animals in the real world. Several algorithms have been proposed to solve the PORL problem [13–16]. These algorithms construct an approximately Markovian state internally from the sequence of past observations, executed actions, and obtained rewards.

Among these, there is an algorithm which solves the PORL problems using an approach based on the free-energy of the stochastic system (e.g., restricted Boltzmann machine: RBM) [15]. This is an extension of Sallans and Hinton’s approach [17] which is able to handle high-dimensional binary states and actions. We call these approaches the free-energy-based reinforcement learning (FERL) framework. Using FERL, sensory information is known to be encoded in the activation patterns of neural populations in a goal-directed fashion. The implementation of this approach in an SNN should provide a top-down glimpse into the neural algorithms of reward-based learning. In this work we propose a SNN implementation of FERL and apply it to the solution of several types of PORL tasks.

This manuscript is organized as follows: First, in the Material and Methods section, we explain FERL and how it may be extended to solve PORL problems. The concepts required for its implementation in SNN, such as pseudo-free-energies, are introduced. Then, in the Results section, we test our SNN model on three tasks with increasing levels of difficulty: a center reaching task (a RL problem), a digit center reaching task (a history-independent PORL problem), and a digit-matching T-maze task (a history-dependent PORL problem). Finally, in the Discussion section, we interpret our results to clarify the remaining issues of this approach and also provide an interpretation of our results from the perspective of biology.

## Methods

### Free-energy-based reinforcement learning

Sallans and Hinton [17] extended the application of the restricted Boltzmann machine (RBM) framework from unsupervised and supervised learning to reinforcement learning. We call their approach of using energy-based modeling in the context of reinforcement learning free-energy-based reinforcement learning (FERL). This is because it uses the free energy of a stochastic system to capture important quantities which appear in reinforcement learning. The RBM is an energy-based statistical model (also known as an “undirected graphical model” or a “Markov random field”) where binary nodes are separated into visible and hidden layers. Nodes in the visible layer are fully connected to nodes in the hidden layer, but there are no connections between nodes within the same layer. Due to this restricted connectivity, given the values of the visible nodes, the posterior distribution over hidden nodes becomes conditionally independent, and it can be computed exactly without heavy computation. In FERL, binary nodes in the visible layer are further classified into state nodes ***s*** and action nodes ***a***.

An energy function of the RBM is given by

$$\begin{array}{c} E\left(s,a,h;\theta \right)=-\sum_{i}\sum_{l}\;s_{i}\;w_{il}^{\mathrm{sh}}\;h_{l}-\sum_{j}\sum_{l}\;a_{j}\;w_{jl}^{\mathrm{ah}}\;h_{l}\;, \end{array}$$ (1)

where $w_{il}^{\text{sh}}\in ℜ$ is the undirected connection weight between a state node *s*_*i*_ ∈ {0, 1} and a hidden node *h*_*l*_ ∈ {0, 1}, and $w_{jl}^{\text{ah}}\in ℜ$ is the undirected connection weight between an action node *a*_*j*_ ∈ {0, 1} and a hidden node *h*_*l*_. Both sets of weights are collectively represented by the parameter ***θ***. Given the energy function, we can also define another important quantity called free-energy. Formally, equilibrium free-energy is the expected energy of the stochastic system at equilibrium minus its entropy:

$$\begin{array}{cc} F(s,a;\theta ) & =\sum_{h}p\left(h|s,a;\theta \right)\;E\left(s,a,h;\theta \right)+\sum_{h}p\left(h|s,a;\theta \right)\;\ln\;p\left(h|s,a;\theta \right) \end{array}$$ (2)

$$\begin{array}{cc} & =-\sum_{i}\sum_{l}\;s_{i}\;w_{il}^{\mathrm{sh}}\;{\hat{h}}_{l}-\sum_{j}\sum_{l}\;a_{j}\;w_{jl}^{\mathrm{ah}}\;{\hat{h}}_{l}+\sum_{l}\left[{\hat{h}}_{l}\;\ln\;{\hat{h}}_{l}+\left(1-{\hat{h}}_{l}\right)\;\ln\;\left(1-{\hat{h}}_{l}\right)\right]\;, \end{array}$$ (3)

where ∑_***h***_ means summation over all possible configurations of hidden nodes ***h***, and ${\hat{h}}_{l}\equiv p(h_{l}=1|s,a;\theta )=\sigma (\sum_{i}w_{il}^{\text{sh}}s_{i}+\sum_{j}w_{jl}^{\text{ah}}a_{j})$ is the probability that node *h*_*l*_ takes the value 1 given the state ***s*** and action ***a*** where *σ*(*z*) ≡ (1 + exp(−*z*))^−1^ is the logistic sigmoid function.

Reinforcement learning algorithms can be optimized by reducing the temporal difference (TD) error between consecutive time steps. If the parameter dependent function approximator $\hat{Q}(s,\;a;\theta )$ with a parameter set ***θ*** is used to estimate the state action value function *Q* ^*π*^(***s, a***) ≡ *E* ^*π*^[∑_*k* = 0_ *γ* ^*k*^ *r*_*k*+1_∣***s***_0_ = ***s, a***_0_ = ***a***], where *E* ^*π*^[⋅] represents the expectation over all possible trajectories produced by using the policy *π*(***s, a***) ≡ *p*(***a**∣**s***) in the given environment, accuracy can be improved after sufficient exploration of the environment if the following SARSA learning rule is used to update the parameters

$$\begin{array}{c} \theta ≔\theta +\alpha \;\left(r_{k+1}+\gamma \;\hat{Q}\left(s_{k+1},a_{k+1};\theta \right)-\hat{Q}\left(s_{k},a_{k};\theta \right)\right)\;\nabla_{\theta }\hat{Q}\left(s_{k},a_{k};\theta \right)\;, \end{array}$$ (4)

where *r*_*k*+1_ is an instantaneous reward obtained after executing an action at step *k*, and *γ* ∈ [0, 1] is the discount factor controlling the influence of the future reward on the value function, *α* is the learning rate set to a small value, and $\nabla_{\theta }\hat{Q}(s_{k},a_{k};\theta )$ is a gradient of the function approximator.

In FERL, the negative free-energy of the RBM, −*F*(***s, a; θ***), is used as an approximator of the state-action value function, $\hat{Q}(s_{k},a_{k};\theta )$, where ***θ*** denotes parameters of an energy function. Therefore the update rule, Eq. (4), can be written as follows:

$$\begin{array}{cc} w_{il}^{\mathrm{sh}} & ≔w_{il}^{\mathrm{sh}}+\alpha \;\left(r_{k+1}-\gamma \;F\left(s_{k+1},a_{k+1};\theta \right)+F\left(s_{k},a_{k};\theta \right)\right)\;s_{i}\;{\hat{h}}_{l}\;, \end{array}$$ (5)

$$\begin{array}{cc} w_{jl}^{\mathrm{ah}} & ≔w_{jl}^{\mathrm{ah}}+\alpha \;\left(r_{k+1}-\gamma \;F\left(s_{k+1},a_{k+1};\theta \right)+F\left(s_{k},a_{k};\theta \right)\right)\;a_{j}\;{\hat{h}}_{l}\;, \end{array}$$ (6)

where $s_{i}{\hat{h}}_{l}$ and $a_{j}{\hat{h}}_{l}$ are the partial derivatives of −*F*(***s***_*k*_, ***a***_*k*_;***θ***) with respect to $w_{il}^{\text{sh}}$ and $w_{jl}^{\text{ah}}$, respectively. This learning rule can be interpreted as follows. When the TD error is positive (good surprise), weights are updated to decrease the free energy of the (***s***_*t*_, ***a***_*t*_) pair so that action ***a***_*t*_ is favored when state ***s***_*t*_ is encountered in the future. On the other hand, when the TD error is negative (bad surprise), weights are updated to increase the free energy of the (***s***_*t*_, ***a***_*t*_) pair so that action ***a***_*t*_ is avoided when state ***s***_*t*_ is encountered in the future. In addition, this update rule has the form of a local Hebbian learning rule modulated by the global TD error.

### Implementation with spiking neuron

#### Leaky integrate-and-fire neuron

Our network is entirely composed of leaky integrate-and-fire neurons [18]. The evolution of the membrane potential of postsynaptic neuron *V*_m_ is given by the ordinary differential equation

$$\begin{array}{ccc} \tau_{m}\frac{dV_{m}}{dt} & = & -\left(V_{m}-V_{\mathrm{rest}}\right)+R_{m}\left(I_{e}+I_{\mathrm{syn}}\left(t\right)+I_{\mathrm{noise}}\right)\;, \end{array}$$ (7)

where *τ*_m_ is the membrane time constant, *V*_rest_ is the resting membrane potential, *I*_e_ is an externally injected current, and *R*_m_ is the membrane resistance. The total synaptic current *I*_syn_ is given by summation over alpha-function *α*(*t*)

$$\begin{array}{ccc} I_{\mathrm{syn}}\left(t\right) & = & \sum_{i}\sum_{t_{s}\in T_{i}}w_{i}\;\alpha \left(t-t_{s}-\delta_{i}\right)\;, \end{array}$$ (8)

$$\begin{array}{ccc} \alpha (t) & = & t/\tau_{\mathrm{syn}}\exp\left(1-t/\tau_{\mathrm{syn}}\right)\cdot H\left(t\right)\;, \end{array}$$ (9)

$$\begin{array}{ccc} H(t) & = & \left\{\begin{array}{cc} 0 & t<0\\ 1 & t\geq 0 \end{array}\right\}\;, \end{array}$$ (10)

where the sum runs over all pre-synaptic neurons *i* and over times *T*_*i*_ of pre-synaptic spikes that a post-synaptic neuron receives after the most recent postsynaptic spike. The amplitude and the synaptic delay of the connection to pre-synaptic neuron *i* are denoted by *w*_*i*_ and *δ*_*i*_, respectively. If the membrane potential *V*_m_ exceeds the threshold *V*_thres_, a spike is generated and *V*_m_ is reset to *V*_reset_:

$$\begin{array}{c} \left\{\begin{array}{cc} x_{i}\left(t\right)=1\;\text{and}\;V_{m}\left(t\right)≔V_{\text{reset}}, & \text{if}\;V_{m}\left(t\right)>V_{\text{thres}}\\ x_{i}\left(t\right)=0, & \text{otherwise}\;, \end{array}\right. \end{array}$$ (11)

where *x*_*i*_(*t*) represents a spike of a neruon *i* at time *t*.

#### Network architecture

**Fig 1:**
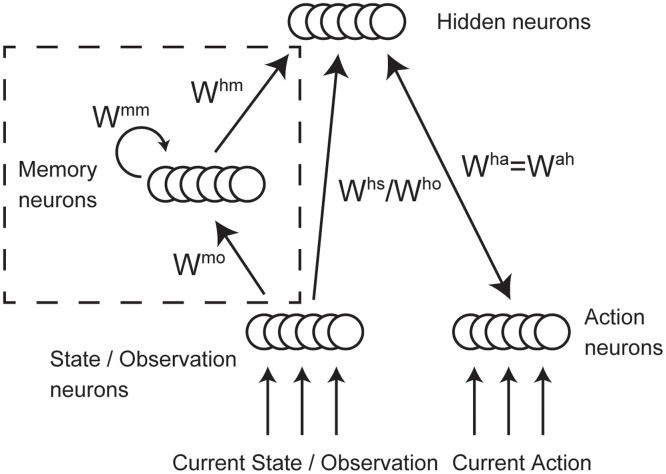
The structures of the spiking neural networks. State neurons are used for the MDP task. Observation neurons are used for the PORL tasks instead of state neurons. Memory architecture (bounded by dashed line in the figure) is introduced only for the history-dependent PORL task.

Binary stochastic nodes in the RBM are replaced by leaky integrate-and-fire neurons in our proposed network model (Fig. 1). The network is composed of state, action, and hidden neurons. The state, by definition, should contain all the information required for optimal decision making. Therefore, in the MDP task, the state layer is composed of state neurons. On the other hand, in the case of history-independent PORL task the state layer is composed of observation neurons, while it is composed of observation and memory neurons in the history-dependent PORL task. When an agent makes an observation or executes an action, neurons associated with the specific observation or action receive direct current. Additionally, all state and action neurons constantly receive noisy input to ensure they operate in a normal firing regime. All observation and memory neurons are unidirectionally connected to all hidden neurons. Action neurons are bidirectionally connected to hidden neurons to reflect the fact that selected actions affect the hidden neurons’ activities.

#### Approximation of free-energy

In order to implement the FERL framework in an SNN, we need to bridge the gap between discrete and continuous time. Let us assume that agent-environment interactions have the following time course. When an agent enters state *s* at discrete time *k*, a set of state neurons ***s*** are activated by current injection. The moment in continuous time this happens at is denoted by $t_{k}^{s}$. Activation of these state neurons ***s*** continues while the agent remains in the current state *s*. Selection of action *a* at discrete time *k* induces the activation of a set of action neurons ***a***. The moment in continuous time this happens at is denoted by $t_{k}^{a}$, ($t_{k}^{s}<t_{k}^{a}$). Activation of these action neurons continues until the next change of state.

In order to include spikes in the framework, we define a time window Δ*t* sufficiently small that it can include at most one spike. Then, the number of spikes within this short interval [*t* − Δ*t, t*) can be described as the ***s***(*t*), ***a***(*t*), and ***h***(*t*) for state, action, and hidden neurons, respectively. During the interval, $\left[t_{k}^{s},t_{k+1}^{s}\right)$, corresponding to the discrete time step *k*, neurons *s*_*i*_(*t*), *a*_*j*_(*t*), and *h*_*l*_(*t*) will be unlikely to take fixed values due to the characteristics of SNN.

Given the formal definition of the free-energy in Eq. (3) and the assumptions that the state nodes *s*_*i, k*_ ≡ [***s***_*k*_]_*i*_ ∈ {0, 1} and the action nodes *a*_*j, k*_ ≡ [***a***_*k*_]_*j*_ ∈ {0, 1} take fixed binary values during continuous time $t\in [t_{k}^{a},t_{k+1}^{s})$, we can introduce a new quantity, “pseudo free-energy for SNN”, as follows:

$$\begin{array}{cc} F(s_{k},a_{k};\theta ) & =-\sum_{i}\sum_{l}s_{i,k}\;w_{il}^{\mathrm{sh}}\;{\bar{h}}_{l,k}-\sum_{j}\sum_{l}a_{j,k}\;w_{jl}^{\mathrm{ah}}\;{\bar{h}}_{l,k}\\ & \;+\sum_{l}\left[{\bar{h}}_{l,k}\;\ln\;{\bar{h}}_{l,k}+\left(1-{\bar{h}}_{l,k}\right)\;\ln\;\left(1-{\bar{h}}_{l,k}\right)\right]\;, \end{array}$$ (12)

where ${\bar{h}}_{l,k}\equiv \frac{1}{N}\sum_{n=1}^{N}h_{l}(t_{k+1}^{s}-n\Delta t)$ is the average firing rate of hidden neuron *l* given the fixed state *s*_*i, k*_ and action neurons *a*_*j, k*_. This quantity has the prefix, “pseudo”, due to the replacement of ${\hat{h}}_{l}$, which is the conditional probability of the RBM’s hidden node *h*_*l*_ to take the value of 1, by ${\bar{h}}_{l}$, which is the average firing rate of the SNN’s hidden neuron *h*_*l*_ defined above. Here $t_{k}^{a}\leq t_{k+1}^{s}-N\Delta t$.

In reality, ***s***_*k*_ and ***a***_*k*_ take stochastic values during time interval $\left[t_{k}^{a},t_{k+1}^{s}\right)$ without injecting extremely strong direct current. Therefore, we define a new quantity, “pseudo-free-energy with average firing rate” (aFE), as follow:

$$\begin{array}{cc} F(I_{s,k},I_{a,k};\theta ) & =-\sum_{i}\sum_{l}{\bar{s}}_{i,k}\;w_{il}^{\mathrm{sh}}\;{\bar{h}}_{l,k}-\sum_{j}\sum_{l}{\bar{a}}_{j,k}\;w_{jl}^{\mathrm{ah}}\;{\bar{h}}_{l,k}\\ & \;+\sum_{l}\left[{\bar{h}}_{l,k}\;\ln\;{\bar{h}}_{l,k}+\left(1-{\bar{h}}_{l,k}\right)\;\ln\;\left(1-{\bar{h}}_{l,k}\right)\right]\;, \end{array}$$ (13)

where ${\bar{s}}_{i,k}$ and ${\bar{a}}_{i,k}$ are the average firing rate of the state neuron *i* and action neuron *j* during the interval $t\in [t_{k}^{a},t_{k+1}^{s})$, respectively. During this interval, the state and action neurons are activated by the current *I*_*s, k*_ and *I*_*a, k*_.

However, the use of average firing rates for the state and action neurons makes the value of the free-energy different from that given by the original definition. Given the fact that we can calculate the instantaneous energy for each time bin, we can also define an average instantaneous pseudo-free-energy (iFE) as follows:

$$\begin{array}{cc} F(I_{s,k},I_{a,k};\theta ) & =-\frac{1}{N}\sum_{n=1}^{N}\{\sum_{i}\sum_{l}s_{i}\left(t_{k+1}^{s}-n\Delta t\right)\;w_{il}^{\mathrm{sh}}\;h_{l}\left(t_{k+1}^{s}-n\Delta t\right)\\ & \;+\sum_{j}\sum_{l}a_{j}\left(t_{k+1}^{s}-n\Delta t\right)\;w_{jl}^{\mathrm{ah}}\;h_{l}\left(t_{k+1}^{s}-n\Delta t\right)\}\\ & \;+\sum_{l}\left[{\bar{h}}_{l,k}\;\ln\;{\bar{h}}_{l,k}+\left(1-{\bar{h}}_{l,k}\right)\;\ln\;\left(1-{\bar{h}}_{l,k}\right)\right]\;. \end{array}$$ (14)

This quantity could be calculated in either a batch or sequential manner. In batch mode, the firing rate of the hidden neurons ***h***_*l*_ are calculated after the system reaches equilibrium. Spikes in 100 ms intervals preceding each observation were used in the calculation. However this approach is not fully plausible biologically since the calculation of the hidden neuron’s average firing rates requires storage of 100 ms spike trains. On the other hand, sequential calculation of the iFE needs only raw spike trains. Let us define the following expression

$$\begin{array}{c} f\left(t;\theta \right)=E\left(s\left(t\right),a\left(t\right),h\left(t\right);\theta \right)+\ln\;p\left(h\left(t\right)|s_{k},a_{k};\theta \right)\;, \end{array}$$ (15)

which resembles the expression appearing in Eq. (12). The first term in Eq. (15) is the energy of the system at time *t* given the neural configuration ***s***(*t*), ***a***(*t*), and ***h***(*t*). The second term is the negative information (also known as “surprise”) associated with spikes ***h***(*t*) given the spikes up to the current moment *t* in the interval $\left[t_{k}^{a},t_{k+1}^{s}\right)$. This second term can be computed sequentially using low pass filtering. In this case, the estimate of the probability *p*(*h*_*l*_(*t*) = 1∣***s***_*k*_, ***a***_*k*_) should be updated according to the following rule

$$\begin{array}{c} {\hat{h}}_{l}\left(t\right)\leftarrow {\hat{h}}_{l}\left(t-\Delta t\right)+\alpha_{h}\;\left(h_{l}\left(t\right)-{\hat{h}}_{l}\left(t-\Delta t\right)\right)\;, \end{array}$$ (16)

where *α*_*h*_ is a small learning rate. Due to our assumption that ***s***_*k*_ and ***a***_*k*_ do not change during the time period $\left[t_{k}^{a},t_{k+1}^{s}\right)$, the second term in Eq. (15) can be approximated sufficiently by tracking this variable for all the hidden neurons. Therefore, *f*(*t*;***θ***) can be approximated by the following expression

$$\begin{array}{c} \hat{f}\left(t;\theta \right)=E\left(s\left(t\right),a\left(t\right),h\left(t\right);\theta \right)+\sum_{l}\left[h_{l}\left(t\right)\;\ln\;{\hat{h}}_{l}\left(t\right)+\left(1-h_{l}\left(t\right)\right)\;\ln\;\left(1-{\hat{h}}_{l}\left(t\right)\right)\right]\;, \end{array}$$ (17)

Then, using Eq. (12) and (17), *F*(***s***_*k*_, ***a***_*k*_;***θ***) can be estimated by Monte Carlo simulation. Since $\hat{f}(t;\theta )$ can be calculated each time step, the accuracy of the estimate of *F*(***s***_*k*_, ***a***_*k*_;***θ***) can now be sequentially improved using only spike data available at the current time *t*. The entire algorithm for the sequential calculation of *F*(***s***_*k*_, ***a***_*k*_;***θ***) using spike data is detailed in Algorithm 1. Both the learning rates *α*_*h*_ and *α*_*f*_ should be appropriately set so that the estimate is smoothly tracked without too much drift.

**Algorithm 1** Sequential estimation of iFE using spike data

**while** $t\in (t_{k}^{a},t_{k+1}^{s}]$ **do**

  Obtain spikes ***s***(*t*), ***a***(*t*), and ***h***(*t*)

${\hat{h}}_{l}(t)\leftarrow {\hat{h}}_{l}(t-\Delta t)+\alpha_{h}(h_{l}(t)-{\hat{h}}_{l}(t-\Delta t)),\;\forall l$

$\hat{f}(t;\theta )=E(s(t),a(t),h(t);\theta )+\sum_{l}\left[h_{l}(t)\ln\;{\hat{h}}_{l}(t)+(1-h_{l}(t))\ln\;(1-{\hat{h}}_{l}(t))\right]$

$\hat{F}(t;\theta )=\hat{F}(t-\Delta t;\theta )+\alpha_{f}\left[\hat{f}(t,\theta )-\hat{F}(t-\Delta t;\theta )\right]$

**end while**

Use $\hat{F}(t;\theta )$ as an estimate of *F*(***s***_*k*_, ***a***_*k*_;***θ***)

### Working memory

In order to solve PORL problems (in particular the matching T maze task described in the results section below) the network should include recurrently connected neurons so that memory of past observations is represented in the network activity. The memory layer is indicated by the dotted box in Fig. 1. The recurrent weights, denoted *w* ^mm^ in Fig. 1, are fixed according to a circular Gaussian distribution. This has the effect of maintaining a characteristic activity pattern across the memory neurons which depends on the past sequence of observations

$$\begin{array}{c} w_{ij}^{\mathrm{mm}}=g_{s}\exp\left(\left(\cos\left(x_{i}-x_{j}\right)-1\right)/g_{w}\right)-g_{b}\;, \end{array}$$ (18)

where *x*_*i*_ is a position of neuron *i* on a circle in radians, *g*_*s*_ is a scaling factor, *g*_*w*_ controls the width of circular gaussian, and *g*_*b*_ biases the average weight.

### Observation

We employed the MNIST dataset (can be downloaded from http://yann.lecun.com/expdb/mnist/) as a high-dimensional observation used in the PORL tasks. The training dataset of the original MNIST dataset was used for feature extraction. We created the training and test sets used for reward-based learning in PORL tasks from the test dataset of the original MNIST dataset. For each dataset, we selected 10 different images for each digit. The size of each image was reduced by cropping all four sides to speed up computation. For the digit center reaching task and the digit matching T-maze task, images are cropped to 22 × 22 pixels and 20 × 15 pixels, respectively. During both training and testing phases in the PORL tasks, digits are randomly selected from corresponding datasets in each time step.

In order to process high-dimensional observations with working memory, the network needs to support both feature extraction and topographically organized activation of a memory layer based on the extracted features. Topographic structures are unlikely to emerge in ordinary RBMs trained using contrastive divergence because this procedure generates maximally independent posterior distributions across the hidden nodes. In order to produce feature extraction and topographic mapping at the same time, the weights between the observation layer and the memory layer were pretrained using the contrastive divergence algorithm (CD-3) [19] with constraints given by the topographic RBM [20]. S1 Fig. describes the activation of hidden nodes during the reconstruction of an observation given a test set of reduced MNIST digits. The weights were trained on a training set of reduced MNIST digits.

### Simulation settings

We used three tasks to test our model: a simple center reaching task as an example of an MDP task, a digit center reaching task as an example of a history-independent PORL task, and a digit matching T-maze task as an example of a history-dependent PORL task. The codes are available gratis at ModelDB (http://senselab.med.yale.edu/modeldb). We used a different number of neurons for each task (Table 1). Network weight were initialized according to a normal distribution with mean 20 and standard deviation 11.88. These parameters were selected so that the initial weights were positive and to ensure that the spiking neurons operated in a normal firing regime.

**Table 1:** The number of neurons in each task.

	Task 1: simple center reaching task	Task 2: digit center reaching task	Task 3: digit matching T-maze task
State / Observation	90	484 (= 22 × 22)	300 (= 20 × 15)
Hidden	90	90	90
Memory			50
Action	90	90	90

All neurons took the same parameters and had the same response properties. The NEST Simulator (http://www.nest-initiative.org) default leaky integrate-and-fire neuron was used in simulations. The membrane time-constant *τ*_m_ was set to 10 ms. The spike threshold *V*_thres_ was set to −55 mV. The resting potential *V*_rest_ was set to −70 mV. The absolute refractory period was set to 2 ms. The reset potential after spikes was set to −70 mV. The membrane capacitance *C*_m_ was set to 250 pF.

Simulations consist of repeated observation-action cycles. One cycle lasts 1000 ms, divided into a 500 ms observation phase and a 500 ms action phase. During the observation phase some state/observation neurons are activated by externally injected current *I*_e_ and an action is selected depending on the activation of action neurons. On the other hand during the action phase both observation and action neurons are activated by input current. The pseudo-free-energy is calculated from the neural activities during the last 100 ms of the action phase.

We used the following parameters for the recurrent weights of the memory neurons in Eq. (18): *g*_*s*_ = 40, *g*_*w*_ = (*π*/72)^2^, and $g_{b}=0.1g_{s}\;\int_{0}^{2\pi }\exp((\cos(x)-1)/g_{w})dx$.

## Results

### Center reaching task

We tested our proposed SNN model of the MDP task, the simple center reaching task, to confirm it works and to compare it to the original RBM. The task includes 7 states, labelled from 0 on the left to state 6 on the right. Agents start at either end of the maze randomly (the states 0 and 6). The middle state 3 is the goal state. Agents can make one of two actions, move one step left (*a* = −1) or move one step right (*a* = 1). When the agent reaches the goal, it receives a large positive reward (*r* = 50,000). All other moves incur small negative rewards (*r* = −1,000). State neurons associated with the current state receive externally injected current *I*_e_ = 1000 pA. Action neurons associated with the selected action and other neurons receive externally injected current *I*_e_ = 1000 pA and *I*_e_ = −2000 pA, respectively. All neurons receive noise current sampled from the normal distribution $I_{\text{noise}}\;\sim \;N(0,600^{2})$ pA.

**Fig 2:**
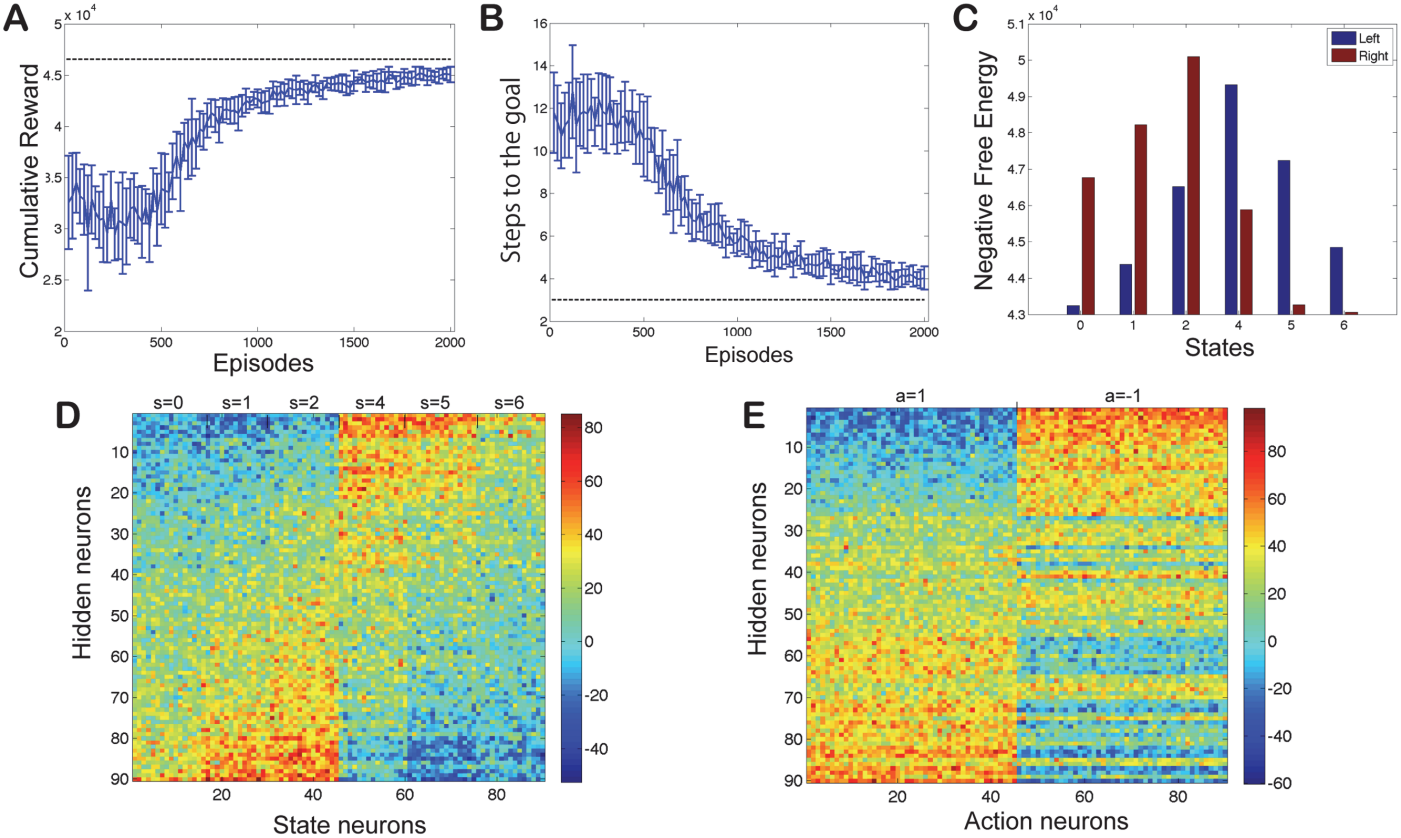
Simple center reaching task. (A–C) Performance of the SNN. (A) The cumulative reward, (B) the number of steps to the goal, and (C) the negative iFE for each state and action (C). (D, E) Connection weights after learning. (D) The weight matrix between the state layer neurons and the hidden layer neurons (*w* ^sh^). (E) The weight matrix between the action layer neurons and the hidden layer neurons (*w* ^ha^). The hidden neurons are sorted by mean weight from the 1st to the 45th state neurons. For states 0, 1, 2, the optimal action is 1, and for states 4, 5, 6, the optimal action is -1.

Fig. 2 describes the performance of an agent in batch update mode. Both cumulative rewards (Fig. 2A) and steps to the goal (Fig. 2B) appear to be approaching their theoretical optimal values of $R_{0}^{*}=47015$ and 3 steps, respectively. The negative iFE (Fig. 2C) properly represents the predicted future reward.

Weights after training are shown in Fig. 2 (D, E). State values appear to be reflected in the weights between the state and hidden neurons, *w* ^sh^, described in Fig. 2D, because the average strength of these weights reflects the number of steps away from the goal. This demonstrates that weights represent state-action values. Weights also appear to encode the goal direction. This is shown by the clear difference between the *w* ^sh^ values associated with the state neurons 1–45 and the *w* ^sh^ values associated with the state neurons 46–90. Similarly the optimal action direction is encoded in the *w* ^ah^ weights shown in Fig. 2E.

**Fig 3:**
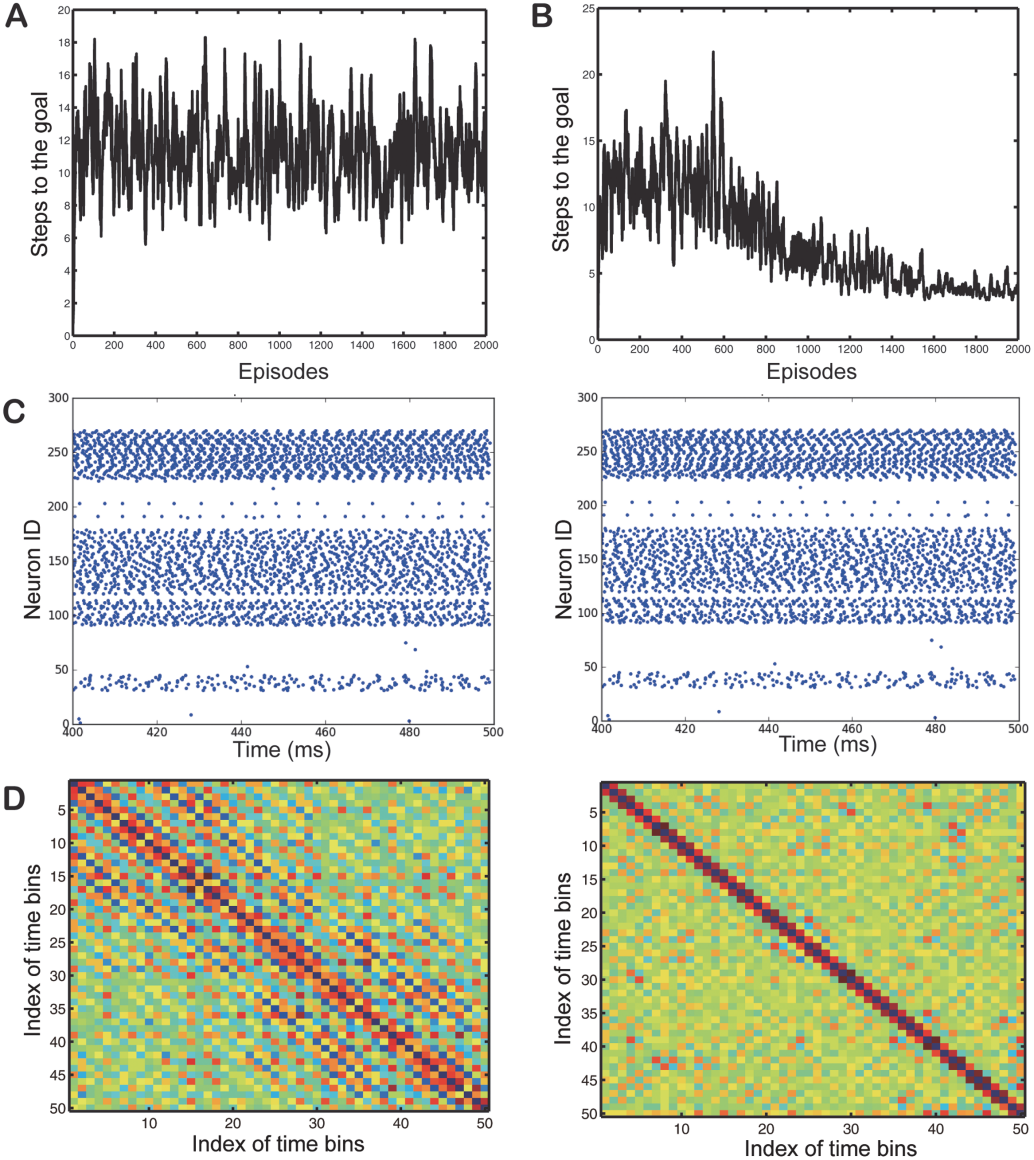
Performance of aFE and iFE. (A) Steps to goal learned with the pseudo-free-energy based on the average firing rate. (B) Steps to goal learned with the average instantaneous pseudo-free-energy under the random delay condition. (C) Firing patterns of all neurons in the random delay condition. Left and right figures use different delays. (D) Distance between the current bin’s firing patterns and those of distant bins (Left: Action neurons, Right: Hidden neurons). The more blue the color the shorter the distance.

In addition to the iFE, we also tested our other approximation to the pseudo-free-energy, the aFE. However, as shown in Fig. 3A, when we use the aFE instead of the iFE, the learning does not occur. One possible explanation for this is that relevant network spiking patterns are lost during the temporal averaging operation before the free-energy computation. To investigate how synaptic delays and spike timing contribute to the pseudo-free-energy, we randomly re-initialized synaptic delays after each episode. Surprisingly we find that learning in the iFE still occurs with synaptic delay randomization. Towards the end of the learning phase, characteristic firing patterns like those shown in Fig. 3 emerge, especially for the action neurons. These firing patterns do not appear when the aFE is used, where they would block learning. Furthermore the firing patterns persist even when the synaptic delays are set to different random values. The presence of firing patterns can be quantified by calculating the distance of neural activations between different time bins during the last 100 ms of the action phase as shown in Fig. 3. As can be seen while both action and hidden neurons show recursive patterns they are much clearer in the hidden neurons where they occur with period around 4–5 time bins.

**Fig 4:**
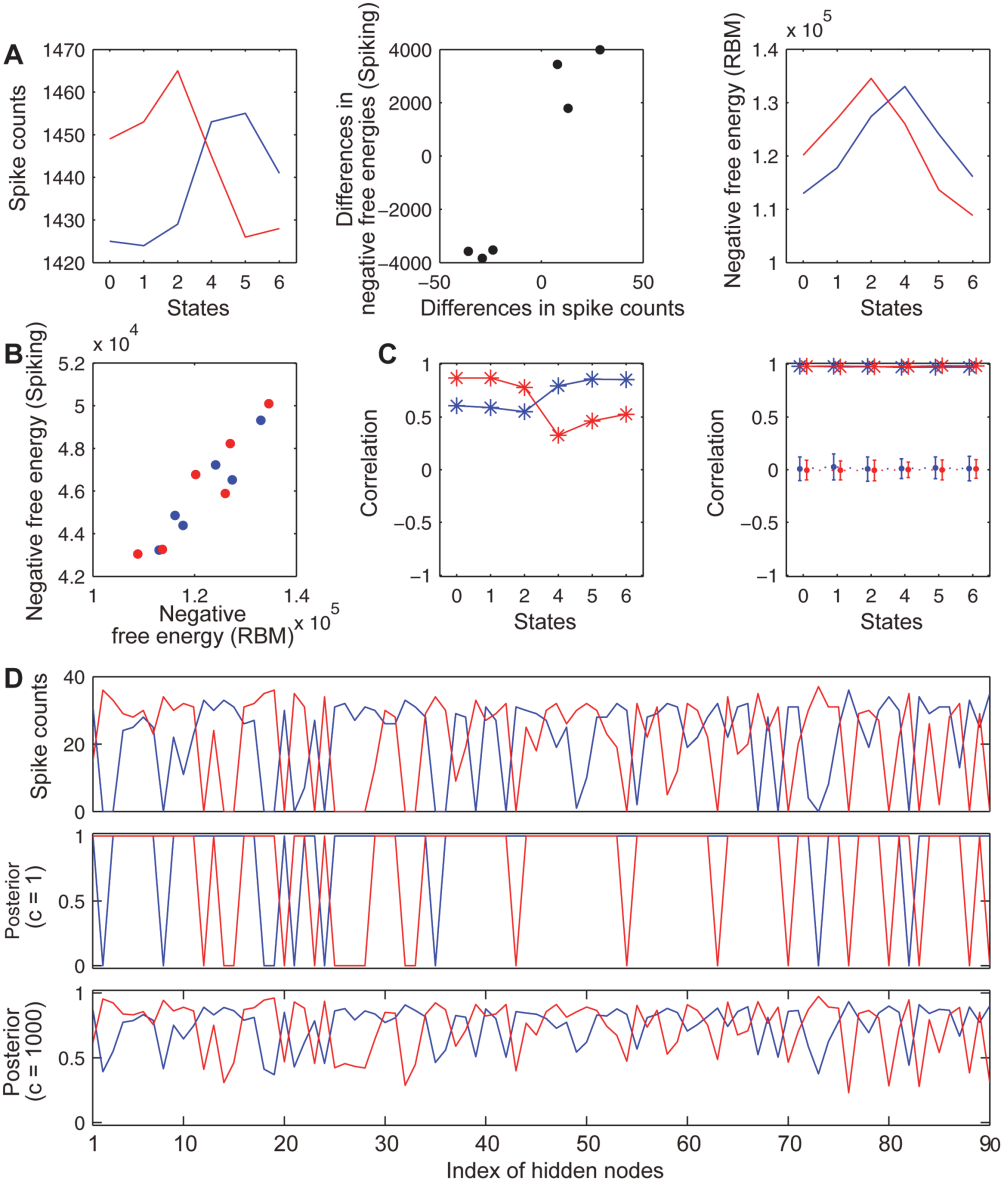
Comparison between the SNN and the original RBM. Red colored symbols and lines indicate rightward actions, blue colored symbols and lines indicate leftward actions. (A) Spike counts of action neurons in the SNN (left) and the negative free-energy in the original RBM (right) for each state. Differences of spike counts (SNN) and negative free-energies (RBM) for action selection (middle). (B) Negative iFE of the SNN and negative free-energy of the equivalent RBM for certain state-action pairs. (C) Correlations between hidden neuron spike counts and the posterior over hidden nodes for each action (left) and when the weights are scaled (right, solid lines) and randomized (right, dotted lines). (D) Spike counts of hidden neurons in the SNN (top panel) and the posterior of the original RBM (middle and bottom panels).

Although it solves the MDP task the performance of our SNN implementation of the RBM is not guaranteed theoretically. Here, we compare our SNN model and the original RBM to determine how functionally different they are (Fig. 4). First we investigate the validity of using spike counts to determine action selection instead of the negative iFE used in the SNN. Spike count “votes” for the two different actions in each of the states are shown in Fig. 4A. These spike counts are determined from the trained network by injecting input current into the associated state neurons for 100 ms. As can be seen by comparing Fig. 2C and Fig. 4A, these spike counts reflect the structure of the negative iFE of the SNN. To quantify how similar the spikes counts and the negative iFE of the SNN are, the negative iFE for the right action is subtracted from that of the left action. This quantity encodes the network preference for left or right action in each state in terms of the iFE. This quantity is more meaningful than the actual iFE value because the relative difference in iFE directly controls the action selection probability along with a globally modulating inverse temperature. It is compared with the analogous quantity calculated using the spike counts in the middle panel of Fig. 4A. The strong correlation between these quantities (r = 0.9621) indicates that spike count based action selection reflects the SNN iFE or equivalently the learned state-action values.

Second, we assess how feasible it is to use an SNN instead of the RBM of the original FERL framework. We construct an RBM using the same weights as the SNN. The right panel in Fig. 4A shows the negative free-energy of the equivalent RBM. Free-energies for certain state-action pairs are calculated by clumping together all associated state-action nodes. Although the free-energies from the constructed RBM are different from the SNN iFE, they are highly correlated (correlation coefficient, *r* = 0.9485) as shown in Fig. 4B. This linear relationship ensures that an action selection probability (or policy) implemented by an SNN can be realized in the equivalent RBM by adjusting the inverse temperature.

To further elucidate the relationship between SNNs and their equivalent RBMs, we compare the activations of hidden neurons of the SNNs and the posterior distributions over hidden nodes of the equivalent RBMs. As shown in Fig. 4C, the correlation between spike counts of hidden neurons and the posterior over hidden nodes is higher for optimal actions than for suboptimal actions in all states. This correlation greatly increases when the SNN weights are divided by 1000 (scaling coefficient, *c* = 1000) to create an equivalent RBM. This increase in correlation is not the general trend observed in RBM with small weights. To clarify this point, we scaled all weights by 1000 after shuffling the state-hidden connection weights and action-hidden connection weights independently. The correlation between the spike counts of hidden neurons and the posterior over hidden nodes vanished after randomly shuffling the weights (20 random shuffles). The correlation was lower in the unscaled condition compared to the scaled condition because the posteriors are saturated in unscaled networks (Fig. 4D, middle). The posterior structure can be observed by scaling the weights and is seen to be very similar to the spike counts (Fig. 4D, top and bottom). Weight scaling is important in creating equivalent RBMs due to the fundamental difference between SNNs and RBMs. Generally speaking, SNNs need to be driven by large weights in the normal firing regime.

S2 Fig. shows the performance of an agent in the sequential update mode. The cumulative rewards appear to approach their theoretical optimal values, in a similar way to the previous result. A sequential estimation of iFE converges at the end. This shows that spikes can be used to sequentially calculate the iFE on the fly.

### Digit center reaching task

**Fig 5:**
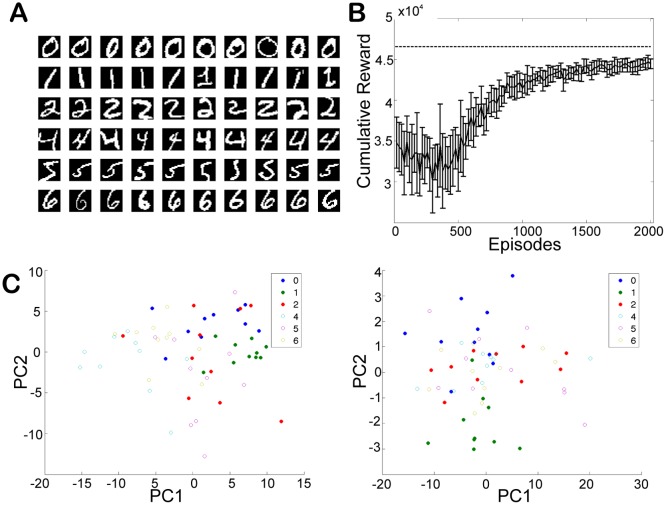
Digit center reaching task. (A) A set of digits used in the training. (B) The cumulative reward obtained with test dataset. (C, D) The activation of hidden neurons projected on the first two principal components in different reward settings. (C) The reward setting is the same as in the simple task. (D) The agent always gets reward of 2000 for any states and actions. Each point shows the hidden activation for each state using test digit dataset.

Next, we test if the proposed architecture solves the history-independent PORL tasks. In this task, observations are stochastic and high-dimensional, but the optimal policy only depends on the true state behind these observations. We employ a task using the same maze as the simple center reaching task, but images of handwritten digits are used. Each of the observation neurons receives input from one of the pixels, so that observation neurons and pixels are in one-to-one correspondence. For the input current to the observation and action neurons *I*_e_ and the noise current *I*_noise_, we employed the same parameter setting used in the previous task. In both training and test phases, at each time step an image is randomly selected from the corresponding dataset, depending on the current state. Even with this high-dimensional input, the agent is able to solve the task (Fig. 5).

Digits with the same optimal action (right action (open circle), left action (close circle)) induce similar activations in the hidden neurons when they are activated by test digit data after learning. As a control we also perform the task under the condition that the agent always gets the same reward for all actions in all states. In this case the activation patterns of the hidden neurons are seen to be independent of reward and clustered according to digit similarity (for example the digit “1” is clustered separately due to its lack of similarity with any of the other digits.)

### Digit matching T-maze task

**Fig 6:**
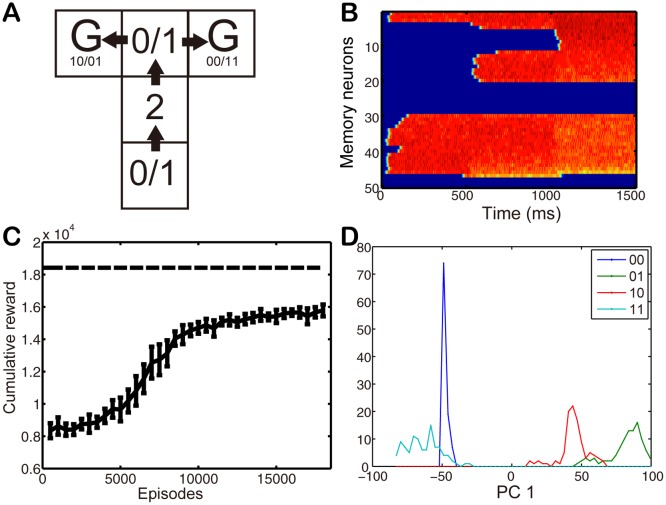
Digit matching T-maze task. (A) Illustration of the task. The agent starts at the bottom-end of the T and makes a decision at the T-junction. The agent observes a “0” or a “1” randomly at these two positions and gets reward if the decision is correct. (B) An example of the activity of the memory neurons. Observations of digits “1”, “2” and “0” are given at 0, 500, and 1000 ms, respectively. The redder the color the higher the neural activity. (C) The cumulative reward. (D) The hidden activity at the decision making position projected on the first principal component. The first principal component accounts for 63.44 percents of the total variance and the second principal component (not shown) accounts for 26.10 percents of the total variance.

Our proposed model is able to solve the two tasks addressed above using only information of the current state or observation. However in the real world it is often the case that tasks cannot be solved solely based on current observations and memory of past experience is required. Therefore we design another task, the digit matching T-maze task (Fig. 6A). This is a history-dependent PORL task using high-dimensional observations. It is a simple extension of a regular T-maze task [21]. In order to act optimally, agents need to use both memory and immediate observation. At the start position and at the T-junction the agent observes one of two randomly chosen digits, “0” or “1”. If the two digits at each end of the central corridor are the same, the agent receives a reward of +20000 at the right goal and reward of −500 at the left goal. On the other hand if the two digits at each end of the corridor are different, the rewards are reversed. The model is extended with the addition of memory architecture. The connection weights from the observation neurons to the memory neurons are pretrained with a topographical RBM [20] in order to obtain different firing patterns in clusters of memory neurons in response to different digits (S1 Fig.). Connections between memory neurons are fixed according to a circular Gaussian distribution so that input dependent activation patterns in these neurons are long-lived. Observation neurons associated with the current state receive externally injected current *I*_e_ = 2000 pA. Action neurons associated with the selected action and other neurons receive externally injected current *I*_e_ = 2000 pA and *I*_e_ = −5000 pA, respectively. All neurons receive noise current sampled from the normal distribution $I_{\text{noise}}\;\sim \;N(0,300^{2})$ pA.

This extended model solves the digit matching T-maze task (Fig. 6). The cumulative reward did not reach its theoretical optimum, which was calculated under the assumption of fully observable state, as shown in Fig. 6C. It is because unlike a fully observable reinforcement learning tasks, in PORL tasks, the agent could not detect the true underlying states and therefore need to construct an approximate internal state with Markovian property, using noisy observations. Hidden neurons show action selection related firing patterns at the decision point which depend on the expected reward rather than on the current position or on past observations.

## Discussion

We constructed a spiking neural network model inspired by the free-energy-based reinforcement learning (FERL) framework. First, we demonstrated that our SNN model had the capacity to handle reinforcement learning tasks in the simple center reaching task. Then we showed that our SNN model could handle high-dimensional input in the digit center reaching task. Finally, we demonstrated that the SNN is able to solve PORL tasks in the digit matching T-maze task. Our results show that FERL can be well approximated by a SNN model.

We have made three contributions in this work. First we proposed an SNN implementation of FERL to solve the PORL problem. Second we made a comparison of SNNs and RBMs. Third we introduced the pseudo-free-energy and its approximations (aFE and iFE) to convert RBMs to SNNs. In this section, we discuss the SNN implementation of FERL and its possibilities.

### 

#### Comparison with the original RBM

First, the biggest difference between the original version of the RBM and the proposed SNN version is the neuron model. The original model uses a binary node, while the proposed model uses a leaky integrate-and-fire neuron model. In the proposed model, due to the fact that the neurons have an absolute refractory period of 2 ms, the maximum number of spikes that can be fired in the 100 ms interval used in the iFE computation is 50. However the actual number of spikes fired is much smaller than this theoretical maximum. For example, after successful learning in the center reaching task, when the agent executed action 1 in state 0, the mean spike counts during the 100 ms iFE computation interval were 14.53 (standard deviation: 0.64) for the state neurons, 31.62 (standard deviation: 2.85) for the hidden neurons, and 34.78 (standard deviation: 0.64) for the action neurons. In the original RBM, state and action nodes are binary, which is equivalent to generate 50 spikes in the SNN during the last 100 ms of the action cycle. Regardless of the low firing rates in the proposed model, the agent is still successful in solving the task.

Second, another difference is discrete and continuous time. This directly influences both action selection and the computation of the free-energy. For the action selection, in order to select an action after an observation is given in the proposed model, spike counts are compared across action neurons. On the other hand in the original RBM the free-energy must be explicitly computed for each action. For the computation of the free-energy, in the proposed model, the average firing rates of the hidden neurons after action selection are used to approximate the posterior distribution, which is computed analytically in the original RBM model.

Third, there are benefits of using continuous time formulation. For the action selection, in our proposed formulation the fact that the firing rate can be computed sequentially means that the variance of the neural firing rate corresponding to a candidate action can be used to control the time the action is executed. For the computation of the free-energy, in the sequential approximation scheme (S2 Fig.), the iFE variance can be calculated on the fly. This quantity represents the current iFE “confidence”. It can therefore be used as an additional variable which controls the learning speed.

#### Learning ability with aFE and iFE

That learning fails with aFE but is successful with iFE despite delay randomization is at first sight puzzling. In this section, we discuss the reasons why learning failed in the case of aFE. One possible reason is that the simulation parameters are not adapted to aFE, which leads to the failure of learning,

Another possible reason is the different firing patterns generated by the aFE and iFE models. Clear firing patterns do not emerge in the aFE model. Learning does not happen in the aFE because firing patterns are smoothed out over the last 100 ms in the action phase, as shown in Fig. 3A. This suggests that firing patterns contribute to learning. The fact that emergent patterns persist in spite of delay randomization provides a clue for the resolution of this puzzle. In our network hidden neurons receive inputs from many action neurons, and vice versa. If presynaptic neurons fire at moderate frequencies postsynaptic neurons tend to accumulate input current at a constant rate regardless of variability in synaptic delays. This persistent input current maintains neurons in a constant firing regime. Also the firing rates of the integrate-and-fire neurons we use in our model saturate as input current is increased. Since neurons have similar firing rates they can generate firing patterns. Also the fact that hidden nodes and action nodes are bilaterally connected ensures that the firing patterns they generate have the same frequency. Recursive firing patterns with the same frequency ensure dominant network configurations, which are represented by a few firing patterns. Therefore, for learning to succeed, the system only needs to learn the same state-action value for these limited firing patterns. This embedding of the same state-action value in the limited but multiple firing patterns ensure the robust maintenance of state-action value and successful learning.

#### Working memory architecture

Our model was able to solve the digit matching T-maze task. This history-dependent PORL task is difficult enough to be considered an approximation to the actual tasks solved by real animals. It requires not only processing of high dimensional input but also depends on retained memories of past observations. Although our model can memorize past states, its architecture does not allow it to discriminate when or how many times the agent has visited any particular state. Furthermore past action sequences are not stored in the current model. Since our main purpose was to propose a neural architecture capable of solving a PORL task, we simply used a memory architecture based on a circular Gaussian distribution. More challenging PORL tasks could be solved by introducing other types of memory architectures [22, 23]. The pretrained weights *w* ^mo^ were also chosen to provide efficient delivery of state information to memory neurons and reduce computational cost. However it might be possible for agents to learn without this weight pretraining if spike timing dependent plasticity or some other learning mechanism is introduced in the recurrent memory networks connections.

#### Biological plausibility

Here we discuss insights into biological reinforcement learning algorithms which can be drawn from our model and its biological plausibility. Since the FERL has been derived on purely theoretical grounds, some points do not agree with the biological evidence. First, the symmetric weight constraint in the RBM is unlikely to be realized in a real biological network. Second, it is difficult to imagine a mechanism whereby TD errors in successive time steps can be computed from the free-energies.

In spite of these inconsistencies with the biological evidence our model has the potential to provide insight into the neural implementation of reinforcement learning. This is because it is able to handle the high dimensional highly noisy observations which are necessary for the solution of PORL tasks in the real world [24]. First, the way sensory inputs are encoded in a goal-directed way in our experiments closely mimics experimental evidence found in the prefrontal cortex [25, 26], the temporal cortex [27], and the lateral intraparietal (LIP) area. As in these experimental studies, activation patterns of our model neurons after reward based learning reflect their reward and action dependent categories (Fig. 5). Second, the FERL update rule appears to be neurally plausible. A wealth of evidence suggests that dopamine encodes the TD error and globally modulates plasticity in striatal neurons [2, 5, 28]. Although our update rule was derived purely from the minimization of a global objective function, the mean squared TD error, it includes not only a global TD term but also a local activity dependent Hebbian learning like term. Third, given the ability in FERL to sample actions according to the policy reflecting the implicitly-encoded learned state-action values, it is possible that biological networks represent state-action values implicitly. Action selection itself is more important than the explicit representation of state-action values. Furthermore analysis of the activation patterns shown by hidden neurons after training in the FERL framework reveals that different neurons encode different types of information such as state-action values and pure state values as well as specific actions [29]. This new perspective provides a new interpretation of experimental results that show the existence of different types of state-action value coding neurons [4]. These characteristics provide a glimpse into underlying laws which are only revealed through a top down approach.

## Supporting Information

S1 Fig Characteristics of the pretrained weights between the observation layer and the memory layer. Weights between the observation layer and the memory layer are trained by the contrastive divergence algorithm (CD-3) [19]. The first (leftmost) column shows the images of hand-written digits (20 × 15 pixels) shown to an agent. The second column shows the posterior over the memory layer given the images of hand-written digits. The third column shows the reconstructed images (observations given corresponding posteriors). The other columns are organized in the same fashion.
(EPS)

S2 Fig Estimation of iFE by sequential manner. The cumulative reward (left). An example of iFE trace during when the agent is one step before the goal and the agent chose the action to reach the goal (right). The each trajectory shows samples before learning (black), during learning (blue), after learning (red).
(EPS)
